# Comparison of Mean Fluoroscopic Time and Mean Contrast Volume Used in Patients Undergoing Coronary Angiography by the Transfemoral Versus Transradial Route

**DOI:** 10.7759/cureus.11700

**Published:** 2020-11-25

**Authors:** Shahzad Hasrat, Safeer Hussain, Zubair Ahmed, Hesham Naeem, Muhammad Sarfraz Khan, Saima Rauf, Abdur Rehman Malik, Adam Umair Ashraf Butt, Areeb Khalid

**Affiliations:** 1 Cardiology, Pakistan Institute of Medical Sciences (PIMS), Rawalpindi, PAK; 2 Cardiology, Rawalpindi Institute of Cardiology, Rawalpindi, PAK; 3 Internal Medicine, Rawalpindi Institute of Cardiology, Rawalpindi, PAK; 4 Internal Medicine: Gastroenterology, Rawalpindi Medical University, Rawalpindi, PAK; 5 Obstetrics and Gynecology, Royal Bolton Hospital, Manchester, GBR; 6 Neurology, Rawalpindi Medical University, Rawalpindi, PAK; 7 Urology, Rawalpindi Medical University, Rawalpindi, PAK; 8 Surgery, Rawalpindi Medical University, Rawalpindi, PAK

**Keywords:** coronary angiography, transradial, transfemoral

## Abstract

Introduction

The prolonged fluoroscopic time during coronary angiography results in a higher radiation dose delivered to patients. Similarly, a higher contrast volume used is associated with higher rates of contrast-induced nephropathy. This study was designed to identify the better technique in terms of lesser fluoroscopic time and volume of contrast used during the procedure.

Objective

To compare mean fluoroscopic time and mean contrast volume used in patients undergoing coronary angiography through the transfemoral versus transradial route.

Methods

A randomized controlled trial (RCT) was conducted at the department of cardiology, Pakistan Institute of Medical Sciences (PIMS) Islamabad between June 2017 and December 2017. Ninety (n=90) patients planned for coronary angiography between 30 and 70 years of age were enrolled. Patients were randomly allocated to Group A (transfemoral route group) and Group B (transradial route group). Fluoroscopic time (minutes) and contrast volume (milliliters) used were measured in each patient.

Results

The mean contrast volume used in Group A was 70.4 ml (SD=8.7) and in Group B, it was 90.1 ml (SD=9.8) (P<0.001). The mean fluoroscopic time in Group A was 5.1 min (SD=1.2), and in group B, it was 8.6 min (SD=1.2) (P<0.001). Similar trends were noted when data were stratified with respect to age and gender.

Conclusion

The mean fluoroscopic time and the mean contrast volume were significantly less in patients where coronary angiography was performed through the transfemoral route than through the transradial route in this study.

## Introduction

Coronary artery disease (CAD) is a leading cause of death and a major cause of morbidity and loss of quality of life, and a leading public health problem accounting for high societal costs [1]. With a growing number of patients suffering from cardiovascular diseases undergoing angiography for the diagnosis of coronary artery disease, the safety of this procedure has become paramount [2]. The transfemoral approach is opted for vascular access during angiography and interventions. This approach is a convenient method for the operator in many ways, as the arterial puncture is usually simple. However, it has been associated with considerable pain, post-procedure hematoma formation, and prolonged hospital stay [3-4]. Compared with transfemoral access cardiac catheterization and percutaneous coronary interventions, using transradial access is associated with lower rates of vascular and bleeding complications, reduced mortality, earlier ambulation, and improved patient satisfaction. However, radial artery spasm is one of the most common access site complications of this procedure, and it occurs in 10%-25% of all cases [5-6]. Other associated disadvantages reported include a longer time of procedure and increased radiation exposure requiring radiation protective methods [7]. In 2013, a study comparing femoral access (mean=142 (SD=39)) and diagnostic coronary angiography via radial access (mean=171 (SD)=72) ml was associated with a higher mean contrast volume (P<0.01). In another recent study conducted in Pakistan comparing transfemoral and transradial routes for coronary angiography (CA), the authors reported that the mean fluoroscopy time was significantly higher in patients who underwent transradial CA (M=6.3(SD=3.8) min) as compared to those who underwent transfemoral CA (M=4.0 (SD=2.9) min). The mean contrast volume used was also higher in patients who underwent transradial (P<0.001) [8].

Angiography is the mainstay for the diagnosis of coronary artery disease and its further management. In our setup, prolonged fluoroscopic times result in higher radiation doses delivered to the patients, and more contrast volumes used are associated with higher rates of contrast-induced nephropathy. Thus, this study will help cardiologists working in tertiary care hospitals identify the better technique in terms of lesser fluoroscopic time and volume of contrast used, minimizing the chances of radiation dose damage and contrast-induced kidney disease in patients indicated for coronary angiography. This study aims to compare the mean fluoroscopic time and mean contrast volume used in patients undergoing coronary angiography through the transfemoral versus transradial route.

## Materials and methods

Study design

A randomized controlled trial (RCT) was conducted at the department of cardiology, Pakistan Institute of Medical Sciences (PIMS), Islamabad, between June 2017 and December 2017, using non-probability consecutive sampling. Patients were randomly allocated to Group A (transfemoral route group) and Group B (transradial route group). Fluoroscopic time (min) and contrast volume (ml) used were measured by well-trained observers in each patient to avoid observer bias.

Sample size

The sample size calculated by using the World Health Organization (WHO) sample size calculator was 90. Table 1 shows the parameters that were kept under consideration during the sample size calculation.

**Table 1:** Parameters for sample size calculation

Parameters	Values
Significance level:	5%
Power of test	90
Population pooled standard deviation	3.35%
Test value of the population mean	6.3
Anticipated population mean	4.0
Sample size for each group	45 per group

Sample selection

Inclusion Criteria

1. All patients planned for coronary angiography; 2. Age 30-70 years; 2. Both genders

Exclusion Criteria

1. History of allergy to iodinated contrast; 2. History of previous coronary artery bypass surgery; 3. History of the previous coronary angioplasty procedure; 4. Abnormal Allen’s test; 5. Pregnant and breast-feeding women

Data collection procedure

The study was started after taking approval from the hospital ethics committee (RTMC Registration no: CRD-2015-042-1124). All patients presenting to the cardiology department of PIMS, Islamabad, planned for coronary angiography were included in the study. Informed written consent was taken from each patient. Initial data about age, contact number, and date of admission were recorded on a predesigned proforma. A detailed history and the clinical examination were recorded before the procedure. The risks and potential benefits of the procedure were explained to the patient. The patients were randomized to either Group A (transfemoral route group) or Group B (transradial route group) by the lottery method. In group A, the procedure was performed through femoral artery puncture and catheterization. In group B, Allen's test was performed to confirm the ulnar artery's patency, followed by radial artery puncture and catheterization. Fluoroscopic time was recorded in minutes, and the volume of contrast injected was calculated and recorded in millimeters by the researcher himself in the prescribed data collection proforma (Annexure I).

Data analysis procedure

The gathered data were analyzed using the Statistical Package for the Social Sciences (SPSS) v.23.0 (IBM Corp., Armonk, NY). Means for quantitative data like age, fluoroscopic time, and volume of contrast were calculated. Frequency and percentages were presented for categorical data. The independent student t-test was used to compare the mean fluoroscopic time and mean volume of contrast used in both groups. Effect modifiers like age and gender were controlled by stratification. The post-stratification t-test was applied, and a P-value < 0.05 was considered statistically significant.

## Results

Demography of the selected population

A total of ninety (n=90) patients of both genders, between the ages of 30 and 70 years planned for coronary angiography, were enrolled in the study. The mean age was 53.60 (SD=10.03) years for the transradial route group and 52.89 (SD=10.27) for the transfemoral route group. Table 2 shows the mean age distribution among groups.

**Table 2:** Mean age distribution SD=standard deviation

Groups	Gender	Mean age (years)	SD
TRANSFEMORAL	MALES	52.64	10.89
	FEMALES	53.20	9.71
TRANSRADIAL	MALES	52.88	10.29
	FEMALES	54.58	9.86

Table 3 shows the frequency and percentage distribution of the demographic profile of the study population.

**Table 3:** Demographic profile of the study population N=number of participants, %=percentage

Variables	Groups	TRANSFEMORAL	TRANSRADIAL	Total
		N (%)	N (%)	N (%)
Gender				
	Male	25 (55.6%)	26 (57.8%)	51 (56.7%)
	Female	20 (44.4%)	19 (42.2%)	39 (43.3%)
Age group				
	30-50 (Years)	19 (42.2%)	15 (33.3%)	34 (37.8%)
	51-70 (Years)	26 (57.8%)	30 (66.7%)	56 (62.2%)

Mean contrast volume and fluoroscopic time in both groups

An independent-samples t-test comparing the mean scores of contrast volume and fluoroscopic time found a significant difference between the means of the two groups t (88)=-13.750, P<.001, and t (88)=-10.088, P<.001), respectively. The mean contrast volume and mean fluoroscopic time were lower in Group A. Mean contrast volume used in Group A was 70.40 ml (SD=8.65), and in Group B, it was 90.09 ml (SD=9.82). Mean fluoroscopic time in Group A was 5.11 min (SD= 1.23), and in Group B, it was 8.64 min (SD=1.21).

Stratification for effect modifiers

Similar trends were noted when data were stratified with respect to age and gender. The mean contrast volume and mean fluoroscopic time were lower in Group A (transfemoral route) patients. Stratified results are shown in Table 4.

**Table 4:** Gender and age-based stratification in both groups for fluoroscopic time (min) and contrast volume (ml) SD=standard deviation

Variables	Groups	FLUOROSCOPIC TIME (min)				MEAN CONTRAST VOLUME (ml)			
		Mean	SD	t(df)	P-value	Mean	SD	t(df)	P-value
GENDER									
MALES				-10.444(49)	< 0.001			-7322(49)	< 0.001
	TRANSFEMORAL	5.12	1.33			69.72	9.08		
	TRANSRADIAL	8.77	1.21			89.58	10.23		
FEMALES				-9.011(37)	< 0.001			-6.881(37)	< 0.001
	TRANSFEMORAL	5.10	1.12			71.25	8.25		
	TRANSRADIAL	8.47	1.22			90.79	9.47		
AGE GROUPS									
30-50 YEARS				-9.418(32)	< 0.001			-7.551(32)	< 0.001
	TRANSFEMORAL	4.79	1.13			67.53	8.04		
	TRANSRADIAL	8.80	1.42			93	11.62		
51-70 YEARS				-10.192(54)	< 0.001			-6.972(54)	< 0.001
	TRANSFEMORAL	5.35	1.26			72.50	8.63		
	TRANSRADIAL	8.57	1.10			88.63	8.64		

## Discussion

The radial route of access is increasingly being used for coronary angiography (CA) and percutaneous coronary intervention (PCI) mainly due to decreased access site bleeding complications, increased patient comfort, and early mobilization [9]. However, concerns have been raised about prolonged procedure time and increased radiation exposure to operators using the radial route of access [10]. However, some studies have shown that with increasing operator experience, radiation exposure can be minimized with a transradial approach. Hence, a majority of radial operators assume that special radiation exposure precautions are unnecessary [11-12]. On the contrary, a few other studies demonstrated increased fluoroscopy time and radiation exposure with radial access and advocated special radiation protection methods to reduce operator radiation exposure, as the amount of radiation exposure in the procedure is far less as compared to a CT scan [13-14]. While this controversy continues, very few studies have compared the operator radiation exposure with the radial versus femoral approaches, particularly in Pakistan. Prolonged fluoroscopic time results in higher radiation doses delivered to the patients and more contrast volumes used are associated with higher rates of contrast-induced nephropathy. The present study was designed to identify the better technique in terms of less fluoroscopic time and volume of contrast used, which would result in reduced radiation dose and reduced rate of contrast-induced kidney disease in patients indicated for coronary angiography. Our results showed that mean contrast volume and mean fluoroscopic time was lower in group A. Mean contrast volume used in Group A was 70.4 ml (SD=8.7), and in group B, it was 90.1 ml ± (SD=9.8) (P<0.001). The mean fluoroscopic time in Group A was 5.1 min (SD=1.2), and in group B, it was 8.6 min (SD=1.2) (P<0.001). Similar trends were noted when data were stratified with respect to age and gender.

Although transradial catheterization is being used more commonly due to increased convenience for the patient, its acceptance among interventional cardiologists is somewhat slow. This is because many of them argue that due to prolonged procedure time and increased radiation exposure, the radial route is not viable for busy catheterization labs. On the other hand, it has been demonstrated that differences between the femoral and radial approaches can be diminished with increased operator experience [15]. Michael TT et al., in their meta-analysis, included 12 studies; of these, five were RCTs (516 participants), and seven (2,808 participants) were registry (observational) studies. One study scored 9 points for quality, and one scored 5; the remaining scored between 6 and 8 (out of a possible 10 points). Follow-up was in-hospital or at 30 days in the majority of studies; one study followed up at nine months [16]. Their results showed that compared with transfemoral percutaneous coronary intervention, fluoroscopy time was higher with radial percutaneous coronary intervention. The radial percutaneous coronary intervention was associated with more frequent access site crossover and shorter hospital stay. When analyzed by study design, the results remained unchanged for non-randomized studies. However, pooling only RCTs resulted in no significant differences between the radial and transfemoral approaches for major adverse cardiovascular and cerebrovascular events (OR 0.65, 95% CI 0.32 to 1.30), death (OR 0.63, 95% CI 0.25 to 1.58), or major bleeding (OR 0.49, 95% CI 0.18 to 1.31). When pooled according to risk, in those studies with more than 30% of participants having a rescue percutaneous coronary intervention, there was no difference in outcomes between the two groups, but in those with less than 30% rescue percutaneous coronary interventions, results were similar to the primary analysis.

In contrast, Farman MT et al. [8] reported that the mean fluoroscopy time was found to be significantly higher in patients who underwent the transradial approach (M=6.3 (SD=3.8)) min and radial-percutaneous coronary intervention (M=15.1 (SD=11.8) min as compared with those who underwent the transfemoral approach (M=4.0 (SD=2.9)) and a femoral-percutaneous coronary intervention (M=10.3(SD=7.4)) with P<.05. Mean fluoroscopy time was also high in the transradial approach. This is understandable if we keep considering the complexity of anatomical variations and technical difficulties. As the authors of the study mentioned, there were about 20 operators, including trainees, fellows, and consultants, who had a marked variation in their experience, skills, and training.

The transradial procedure has been proven to be cost-effective in terms of using a limited number of catheters [17]. Transradial diagnostic coronary angiography can be done with one multipurpose catheter. In contrast, a transfemoral coronary angiogram needs at least two and usually three catheters. Anatomical variations (atypical anatomy), such as tortuous configurations, stenoses, hypoplasias, and radioulnar loops, are commonly encountered during the transradial approach for diagnostic and interventional procedures and may cause access failure [18]. Lo et al. [19] recently studied 1,540 consecutive radial procedures and found a radial artery anomaly in 13.8% of patients while Valsecchi et al. [20] reported quite a high incidence of 22.8% in his study of 2,211 cases. 

It is believed that fluoroscopy time can be minimized with increased experience, particularly in the transradial approach, where a significantly large difference in fluoroscopy use was noted among experienced and inexperienced groups. Recently, Weaver et al. [21] compared the transradial approach versus transfemoral approach in patients presenting with STEMI and reported significantly less fluoroscopy use in transradial approach (M=12.5 (SD=7.9) versus M=15.2 (SD=10.1) min; P=0.02). Similarly, Rathore et al. reported no significant difference in the length of fluoroscopy time when comparing the transradial approach with the transfemoral approach in patients who underwent percutaneous coronary intervention for chronic total occlusions [22]. A study focusing on the post-procedural complications associated with these techniques, with larger sample size, is recommended.

## Conclusions

The mean fluoroscopic time and the mean contrast volume were significantly less in patients where a coronary angiography was performed through the transfemoral route than through the transradial route in this study. Further controlled trials are needed to adopt a transfemoral route for coronary angiography in routine clinical practice.
